# Organizing and Analyzing Data from the SHARE Study with an Application to Age and Sex Differences in Depressive Symptoms

**DOI:** 10.3390/ijerph18189684

**Published:** 2021-09-14

**Authors:** Lara Lusa, Marianne Huebner

**Affiliations:** 1 Department of Mathematics, Faculty of Mathematics, Natural Sciences and Information Technologies, University of Primorska, 1000 Koper/Capodistria, Slovenia; 2 Medical Faculty, Institute for Biostatistics and Medical Informatics, University of Ljubljana, 1000 Ljubljana, Slovenia; 3 Department of Statistics and Probability, Michigan State University, East Lansing, MI 48824, USA; huebner@msu.edu

**Keywords:** SHARE, initial data analysis, data cleaning, prediction modelling, depression, older adults, sex differences, Europe

## Abstract

The SHARE study contains health, lifestyle, and socioeconomic data from individuals ages 50 and older in European countries collected over several waves. Leveraging these data for research purposes can be daunting due to the complex structure of the longitudinal design. The two aims of our study are (1) to develop a framework and R code for data management of the SHARE data to prepare for data analysis, and (2) to demonstrate how to apply the framework to a specific research question, where the aim is to model the presence of clinically significant depression assessed by the 12-item Europe depression scale. The result is a framework that substantially reduces the time to initiate research studies using SHARE data, facilitating the data extraction, data preparation and initial data analysis, with reproducible R code. Further, we illustrate the extensive work required to prepare an analysis-ready data set to ensure the validity of the modeling results. This underlines the importance of carefully considering and recording data management decisions that have to be built into the research process. The results about sex differences in the probability of depression are consistent with previous literature. Our findings about age-associated changes can be opportunities for adequate treatment interventions.

## 1. Introduction

The Survey of Health, Ageing and Retirement in Europe (SHARE) is a multinational panel data survey, collecting data on medical, economic and social characteristics of about 140,000 unique participants after age 50, from 28 European countries and Israel [1]. The intent is to collect information on health and socio-economic factors for an aging population, using a longitudinal study design ’to allow insights in the fields of public health and socio-economic living conditions of European individuals’ (http://www.share-project.org [1] accessed on 15 July 2021). The data are harmonized across countries as well as with other studies in aging such as the U.S. Study of Health and Retirement (HRS) [2] and the English Longitudinal Study of Ageing (ELSA) [3] and thus is an extraordinary resource. Data collection started in 2004 and takes place approximately every two years. Most of the waves focus on the current living circumstances of the participants, while others address specific issues, such as retrospective life histories or COVID-19. The dataset derived from the SHARE study is publicly available for scientific purposes after registration, in Stata [4] and SPSS [5] formats; a simplified version of the dataset called easySHARE can be used for teaching purposes and can be distributed to students and is provided also in R format [6]. The data is organized in 25 modules and comprise about 1000 questions, with a complex structure. For example, the baseline and the longitudinal questionnaires differ in some aspects, and questions changed during the course of the study. Despite the great effort in ensuring cross-national harmonization, some differences between countries exist, since (1) some countries were included only in some waves; (2) the sampling designs vary across countries and are based on random or stratified sampling; (3) new participants (refreshment samples) are included using different strategies in different countries; (4) countries can use sub-questionnaires; (5) in some countries SHARE is linked to national mortality data. The details are described in [1] and extensive documentation and details on meta-data are provided on the web site of the project http://www.share-project.org/data-access.html accessed on 15 July 2021.

Despite the public availability and excellent documentation, a non-trivial amount of background information needs to be carefully considered before developing a valid and feasible statistical analysis plan for a research aim based on SHARE data. The retrieval and linking of SHARE data require expertise in data management. A thorough understanding and inspection of data properties is needed to assure correct interpretation of findings of prediction models. To overcome some of these challenges, a STATA module was developed for a partial automation of data download and pre-processing [7]. However, the use of the module requires a valid STATA licence and familiarity with the program.

We developed functions written in open source statistical software R [6] for data download, data pre-processing, and data management for SHARE data to facilitate the selection of variables within modules and waves, and their retrieval in R or text format. The downloaded data can then be analyzed using the R programming language or any other statistical software.

The first aim of this paper is to present a framework that substantially reduces the time to initiate research studies using SHARE data, facilitating the extraction of data, data preparation and initial data analysis, providing reproducible R code. The second aim is applying this framework in a case study on clinical depression in older adults. In this application we develop a predictive model to estimate the probability of depression for known risk factors and examine age-associated changes for men and women and regional differences in European countries, using cross-sectional data. Depression is a major cause of disability especially at older ages and there are sex differences [8]. The 12 item Europe Depression scale (EURO-D) was specifically developed for older people and validated in several countries. Clinically significant depression is defined with a EURO-D score of 4 or higher [9,10]. Contributing factors to depression are lower education level, presence of chronic diseases, lack of physical activity and other life-style variables. This case study illustrates the challenges in examining data quality for longitudinal data collections and preparing analysis-ready data sets by measurement occasions, including cross-sectional studies with only one measurement occasion.

The paper is organized as follows: In the Materials and Methods section we describe the SHARE study and data management to prepare the data for analysis in a research study. We illustrate the use with a data application where we assess the association of age, sex, geographic region and other known risk factors with the presence of clinically significant depression using cross-sectional data and logistic regression models. In the Results section we show aspects of data cleaning and discuss age-associated patterns of predicted probabilities of depression. We emphasize the importance of initial data analysis and the impact of statistical modeling choices in the Discussion.

## 2. Materials and Methods

### 2.1. SHARE Data

SHARE data are publicly available for scientific purposes after registration at http://www.share-project.org/data-access.html accessed on 15 July 2021, only users without a scientific affiliation need to provide a research plan (detailed terms of use are listed at http://www.share-project.org/data-access/share-conditions-of-use.html accessed on 15 July 2021). The SHARE study is subject to continuous ethics review. The continuation of the project was reviewed and approved by the Ethics Council of the Max-Planck-Society. The target population are all individuals who are at least 50 years old, speak the official language of the country, do not live abroad or in an institution such as a prison, or their spouse or partner, independent of age. A target population also consists of households with at least one member satisfying the conditions listed above [11]. Data are stored in ZIP files and can be categorized in three types: (i) Data from specific waves (folder names starting start with sharew, for example sharew1 for wave 1); (ii) data across waves (where folder names start with sharewX and include longitudinal weights, coverscreen (CV_R) data, job episodes panel, linkage data); (iii) a simplified data set for teaching purposes, easySHARE data with 109 variables. The data are organized in 25 modules and comprise about 1000 questions (Table A1) regarding demographics, occupation, income, health, life-style and health behaviors. Further explanations about the data organization and the modules are in Appendix A. The sampling strategy is country specific (stratified simple random sampling or multi-stage sampling), the selection unit was the household (with at least one person aged 50 or older).

Missing values are coded with negative values that describe the reason or type of missingness (such as −1: ’Don’t know’, −2: ’Refusal’, −3: ’Implausible value/suspected wrong’, −9: ’Not applicable’). Five multiple imputations are provided for a selected set of variables with item non-response, using hot-deck imputations or fully conditional specification method in the cross-sectional module gv_imputations (not provided for SHARELIFE interviews, see methodological details in [12]).

Data can be downloaded in STATA or SPSS format. An R function for the data import is described in Appendix B.

### 2.2. SHARE Data Management

Data management for research projects using SHARE data are also common in a range of other research studies and other data. This includes

- data filtering of participants or interviews based on inclusion/exclusion criteria specified in the statistical analysis plan
- management of missing values including data cleaning tasks, such as elimination of inadmissible values, checking for consistency of values
- variable management, for example renaming, deriving new variables by aggregating levels or values from different variables, or by using definitions that are specific to certain waves or types of interview
- formatting or subsetting datasets for different analyses, such as baseline or first interview data or datasets ordered by wave or measurement occasion for longitudinal projects.

We developed reproducible R code for a structured process to create an analysis ready data set, and for conducting a systematic initial data analysis [13]. This empowers researchers to use this rich resource and efficiently work with the SHARE data.

### 2.3. Data Application: Age-Associated Patterns of Depression and Sex Differences

#### 2.3.1. Study Population

**Figure 1:**
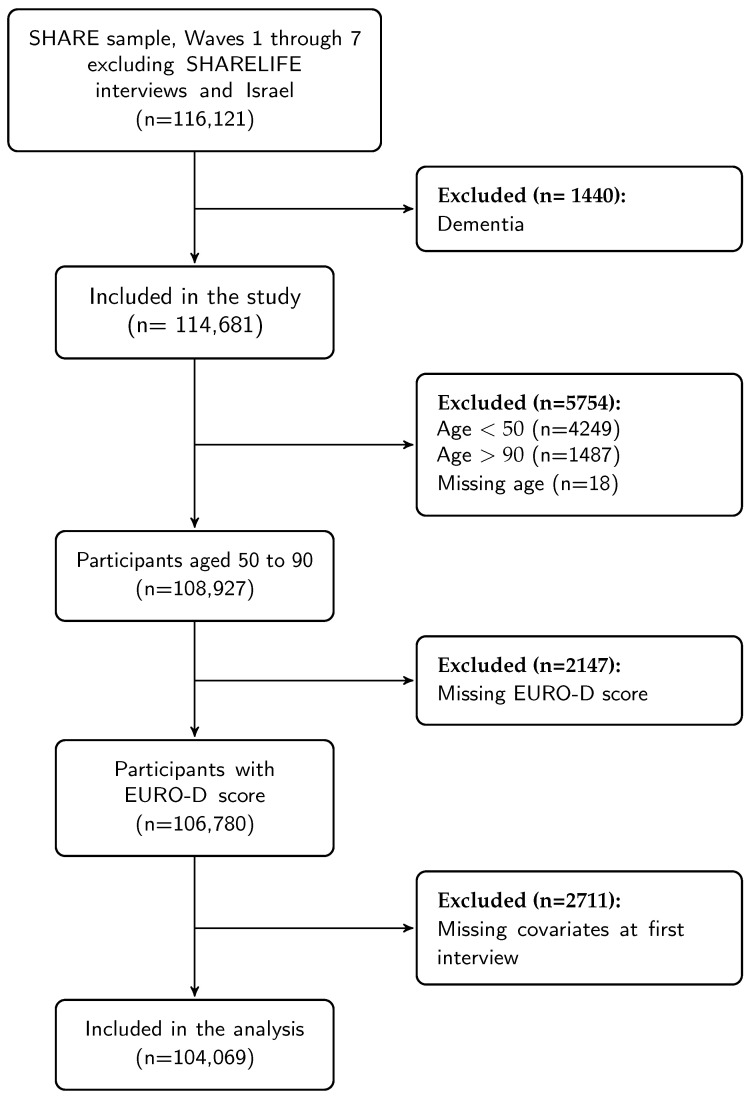
Study flow diagram.

This cross-sectional study on depression includes men and women aged 50 to 90 from 20 European countries and excludes countries with only SHARELIFE interviews. Demographic, health, and life-style variables at the first interview were obtained from Waves 1 through 7. Exclusion criteria were unknown birth date, dementia, or missing EURO-D score. The sample sizes are shown in the study flow diagram (Figure 1), the number of observations by region in each wave are presented in Table 1.

#### 2.3.2. Outcome Variable—Depressive Symptoms

Depressive symptoms were assessed in SHARE using the EURO-D scale, which was validated in several countries [9,10]. Each symptom of the 12 EURO-D scale (depressed mood, pessimism, wishing death, guilt, sleep, loss of interest, irritability, loss of appetite, fatigue, poor concentration, lack of enjoyment and tearfulness) is scored 0 (symptom not present) or 1 (symptom present), thus generating an ordinal scale with a range of scores from 0 to 12. Higher scores represent more severe depression, clinically significant depression is defined with a EURO-D score of 4 or higher [10].

#### 2.3.3. Explanatory Variables

Variables used in the analysis encompass demographics, chronic diseases, lifestyle factors and health behaviors.

- Demographics. Variables include: age, sex, highest education level, region of residence. Educational level was assessed as self-reported highest educational attainment based on the ISCED 1997 classification and recorded into low (ISCED groups 0–2), medium (ISCED groups 3–4), high (ISCED groups 5–6) or other. Countries of residence were classified into four regions: Northern Europe (Denmark and Sweden), Western Europe (Austria, Germany, France, the Netherlands, Switzerland, Belgium, Ireland and Luxembourg), Southern Europe (Spain, Italy, Greece and Portugal) and Eastern Europe (Czech Republic, Poland, Hungary, Slovenia, Estonia and Croatia).
- Body mass index. Self-reported height and weight were converted into body mass index (BMI).
- Chronic diseases. Information on chronic diseases was gathered as response to the question, ‘Has a doctor ever told you that you had any of the following conditions?’ and included 6 conditions (cancer, chronic obstructive pulmonary disease (COPD), coronary heart disease, diabetes, hypertension and stroke) following a consistent approach to measuring occurrence of chronic conditions [14]. The responses were summarized as having 0, 1, or 2+ conditions.
- Life-style factors and health behaviors. Variables included living arrangements, current smoking, physical activity, and instrumental activities of daily living. Physical activity was analyzed as an ordinal scale with vigorous physical activity more than once a week, or one to three times a month, or once a week, or hardly ever or never. The instrumental activities of everyday life (IADL) index is constructed across individual’s difficulty doing each of the following everyday activities [15]: ‘doing work around the house or garden’, ‘leaving the house independently/accessing transportation’, ‘shopping for groceries’, ‘doing personal laundry’, ‘managing money’, ‘preparing a hot meal’, ‘taking medications’ and ‘making telephone calls’. Individuals were instructed to exclude any difficulties expected to last less than three months. This variable was dichotomized as follows: ‘no IADL limitation’ and ‘one or more IADL limitations’. Current smoking was a binary variable. Living arrangement was dichotomized as follows: or ‘not living alone’.

#### 2.3.4. Statistical Analysis for Modeling Depression

We used a binary outcome variable (presence of clinical depression defined by the EURO-D score ≥4) and modelled it using logistic regression, which is a commonly used statistical regression model for dichotomous outcome variables, including in studies on depression. Logistic regression allows the estimation of the probability of experiencing an event on the basis of the values of multiple explanatory variables; we used it to evaluate the probability of clinical depression and its dependence on several known risk factors. More specifically, we evaluated the association of age, sex, geographic regions and all their 2-way interactions on the occurrence of clinical depression. This is referred to as the ’base model’. We used two-way interactions because we expected that the effect of age and sex may vary across geographical regions [16], and that sex differences could change at older ages [8].

Inclusion of additional variables was guided by risk factors for depression identified in previous studies [17,18] and comprised education, BMI, current smoking, presence of chronic disease, vigorous physical activity, living arrangement; in the following these models are referred to as adjusted models. Restricted cubic splines (rcs) [19] with 5 knots were used to flexibly model the association between age and probability of depression, thus avoiding apriori assumptions of linearity (on the logit scale) between age and outcome. Age was centered at 50 years, BMI was scaled in steps of 10 units. We compared these models to models with a linear term for age. Results from base and adjusted models were presented as odds ratios (OR) and 95% confidence intervals (CI). The estimated probability of depression (with 95% pointwise CI) across ages was presented graphically in different regions and by sex. These models were then fitted to each of the 12 symptoms that make up the EURO-D score.

We performed a sensitivity analysis to evaluate the effect of not using sampling weights in the regression analyses on depression. The sensitivity analysis consisted in comparing the results from weighted and unweighted analyses within single waves.

All analyses were conducted with the statistical software R (version 4.0.2) [6].

## 3. Results

### 3.1. SHARE Data

SHARE data have already been checked for data quality and data cleaning steps have been performed, but a workflow for data preparation prior to analysis including a data inspection for admissible values, consistency across repeated measurements, and data properties is advisable [13].

- Number of participants and time metrics. The number of participants can be summarized by wave or first interview (Table 1). The provided R code facilitates the application of exclusion criteria with resulting changes in numbers at each step, e.g., Figure 1. Other time metrics, such as measurement occasion, were defined to facilitate data summaries for number of participants or baseline characteristics. Furthermore, it is also possible to extract information about an individual’s progression throughout the data collection process, for example intermittent participation.
- Missing values. The workflow [13] includes stratifying missing values by wave and questionnaire to identify potential issues for each variable. This made it possible to reduce the number of missing values.
  - Marital status had a large proportion of missing data, and the stratification of missing values by type of questionnaire (baseline or longitudinal) showed that this item was missing mostly in longitudinal questionnaires, because the value was recorded only if changes from previous interviews occurred. This information allowed us to define a more complete variable.
  - Chronic obstructive pulmonary disease (COPD) was defined as the combination of the answer to two questions in waves 1 and 2 (Selected for questions ph006d6 (Chronic lung disease such as chronic bronchitis or emphysema) or ph006d7 (asthma) from the PH module), while only one question ph006d6 was used from wave 4, which already included asthma.
  - The EURO-D score, defined as the sum of selections of 12 symptoms, and the dichotomized version to indicate clinical depression (EURO-D ≥ 4) are available in the gv_health module. These variables were missing if any of the 12 symptoms was missing, thus 7790 interviews had missing EURO-D (out of 258,207). However, 30% of the clinical depression indicator variables could be retrieved based on the available symptoms thus reducing missingness.
  - Current smoking in the SHARE module gv_health:cusmoke is missing in all interviews after wave 5. However, smoking status at baseline interviews can be retrieved using responses to questions ‘Have you ever smoked?’ (br:br001_ ) and ‘Are you currently smoking?’ (br:br002_). This removes most of the missing values of the smoking indicator from the baseline interviews and more than 50% of the missing values in the longitudinal interviews.
  - Education is recorded only at baseline interviews (ISCED 1997), but its value is reported also in the other waves through the generated variables in the gv_isced module. Replacing missing values based on the first non-missing value reduced missingness by 40% (from 2855 to 1720 interviews).
  - Assuming that height does not vary greatly over time we replaced missing values at first interview with the first available measurement. This also reduced the number of missing values of BMI.

Such tasks are part of a systematic process of initial data analysis (IDA, [20]). It facilitates evaluating data properties in baseline and longitudinal questionnaires before applying statistical models to the data.

### 3.2. Data Application: Depression in Older Adults

The study included 104,069 participants from 20 countries. The distribution across geographic regions was Western (41%), Eastern (26.4%), Southern (21.9%) and Northern (10.8%). Participant characteristics, stratified by sex and region are described in Table 2. The age distribution was similar across sex and geographic regions with fewer participants at older ages. Compared to other regions residents of Southern European countries had lower education and engaged less in vigorous physical activities. Residents of Northern European countries had higher education levels and engaged more in vigorous physical activities. Comorbidities and IADL limitations were more common in the Eastern region. Compared to men, women smoked less, had lower education, were less often engaged in vigorous physical activity, and  more likely to live alone. These differences were less pronounced in the Northern region.

Depression defined by the EURO-D score was more common in women than men (34% vs. 19%). The prevalence was lower than average in the Northern region and above the average in the Southern and Eastern regions.

**Figure 2:**
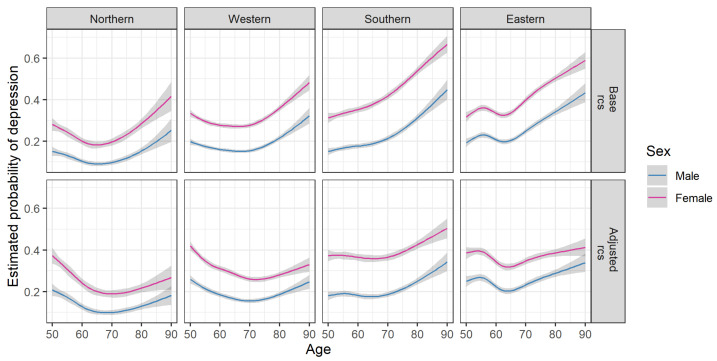
Estimated probabilities of depression by age, sex and region. Estimates were obtained from logistic regression models including two-way interactions between age, sex and region (base model) and additional risk factors (education, BMI, current smoking, presence of chronic disease, vigorous physical activity, living arrangement; adjusted model); age was modelled using restricted cubic splines (rcs). See Table 1 for sample sizes within each region and by sex, and for descriptive statistics.

**Figure A1:**
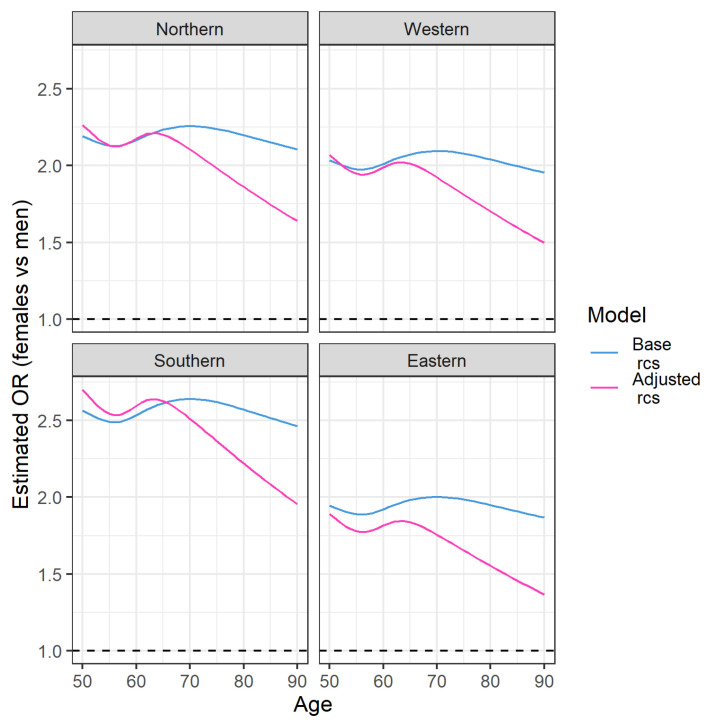
Sex differences by age and region in depression; OR were obtained using logistic regression models including two-way interactions between age, sex and region (base model) and additional risk factors (education, BMI, current smoking, presence of chronic disease, vigorous physical activity, living arrangement; adjusted model); age was modelled using restricted cubic splines (rcs). See Table 1 for sample sizes within each region and by sex, and for descriptive statistics.

Predicted probabilities of depression are shown in Figure 2. The base model included two-way interactions between age, sex and region while the adjusted model included further risk factors for depression. Women had considerably higher probability of depression at all ages and in all regions (Appendix C Figure A1). The age-associated pattern in depression differed by geographic region but the estimates for men and women within regions were very similar.

After adjusting for risk factors the probability of depression decreased between 50 and 70 years (from 0.4 to 0.2 for women and from 0.2 to 0.1 for men) in the Northern and Western regions. In the Southern region the probability of depression remained stable between ages 50 and 70, followed by a steeper increase than in other regions and there was a larger difference between men and women. In the Eastern region there was a decrease in the probability of depression between ages 55 and 65 followed by an increase. Without adjusting for risk factors the age-associated patterns were similar, but there was a more pronounced rise in the probability of depression after age 70.

The sex differences in odds ratios for depression decreased after age 65 in the adjusted model (interaction term between sex and age *p* = 0.0025, Appendix C Figure A1). Risk factors of depression were lower education, smoking, chronic diseases, living alone, and lack of vigorous physical activity (Table 3). Sensitivity analyses indicated that the association between risk factors and depression was similar for weighted and unweighted analyses. Details are given in Appendix D.

**Figure A2:**
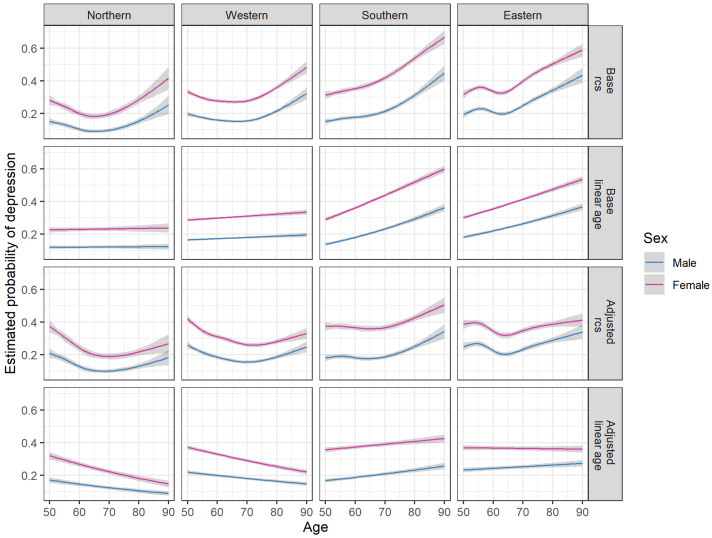
Comparison of the estimated probabilities modeling age with restricted cubic splines (rcs) or linearly. Estimates were obtained using logistic regression models including two-way interactions between age, sex and region (base model) and additional risk factors (education, BMI, current smoking, presence of chronic disease, vigorous physical activity, living arrangement; adjusted model); age was modelled using restricted cubic splines (rcs) or linearly (linear age). See Table 1 for sample sizes within each region and by sex, and for descriptive statistics.

The choice of functional form and the inclusion of risk factors in the statistical models may account for differences in conclusions in various studies. Age-associated patterns of depression differ for linear effects of age compared to non-linear effects. For example, in the Northern region depression did not change with age in a linear model in contrast to models with nonlinear age effects (Appendix C Figure A2). However, there was a decline in depression with age in linear models adjusted for risk factors.

### 3.3. Analysis of Depressive Symptoms

**Figure 3:**
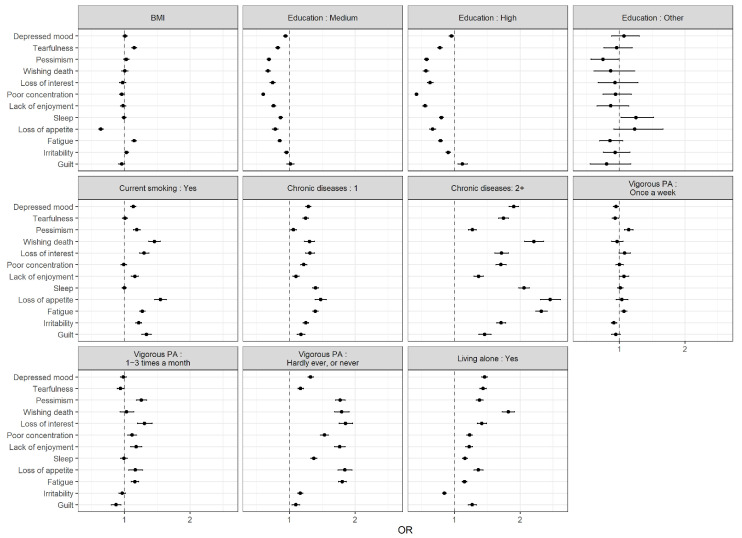
Factors associated with depressive symptoms (OR and 95% CI). Estimates were obtained from 12 separate logistic regression models (one for each symptom) including two-way interactions between age, sex and region and additional risk factors (education, BMI, current smoking, presence of chronic disease, vigorous physical activity, living arrangement ); age was modelled using restricted cubic splines (rcs). See Table 3 for sample sizes within each region and sex and for descriptive statistics.

Risk factors for depression were also associated with single depressive symptoms (Figure 3). Higher education was associated to lower probability of reporting several symptoms, while presence of chronic diseases or lack of vigorous activities increased the risk of depressive symptoms.

**Figure 4:**
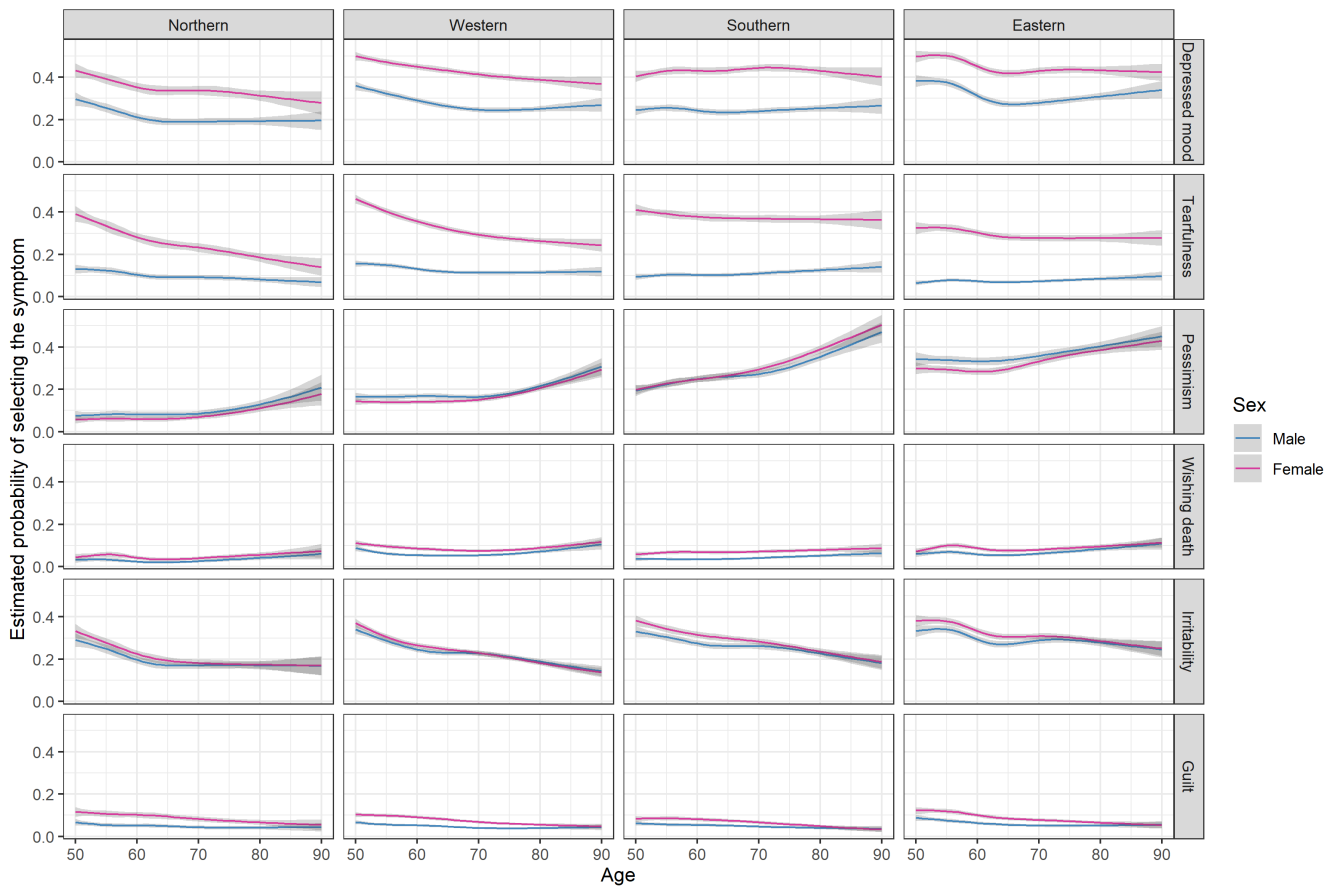
Estimated probability of selecting a symptom (affective, irritability and guilt). Estimates were obtained from logistic regression models including two-way interactions between age, sex and region (base model) and additional risk factors (education, BMI, current smoking, presence of chronic disease, vigorous physical activity, living arrangement; adjusted model); age was modelled using restricted cubic splines (rcs). See Table 3 for sample sizes within each region and by sex, and for descriptive statistics.

**Figure 5:**
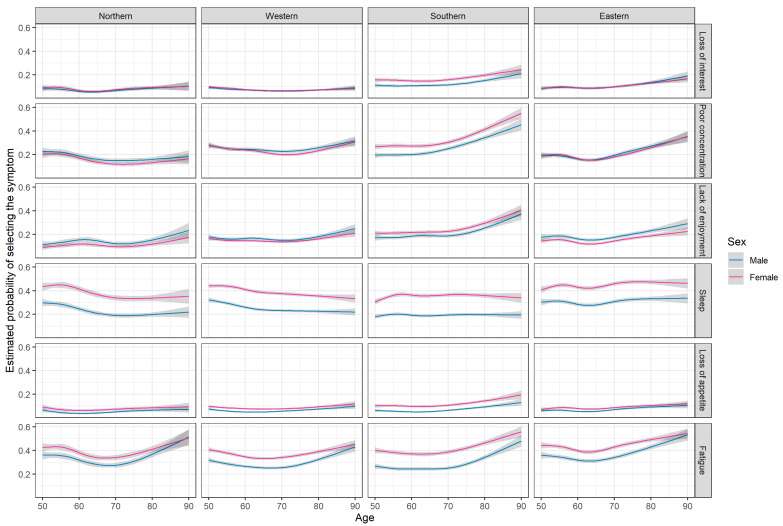
Estimated probability of selecting a symptom (motivation and somatic). Estimates were obtained from logistic regression models including two-way interactions between age, sex and region (base model) and additional risk factors (education, BMI, current smoking, presence of chronic disease, vigorous physical activity, living arrangement; adjusted model); age was modelled using restricted cubic splines (rcs). See Table 3 for sample sizes within each region and by sex, and for descriptive statistics.

**Figure A3:**
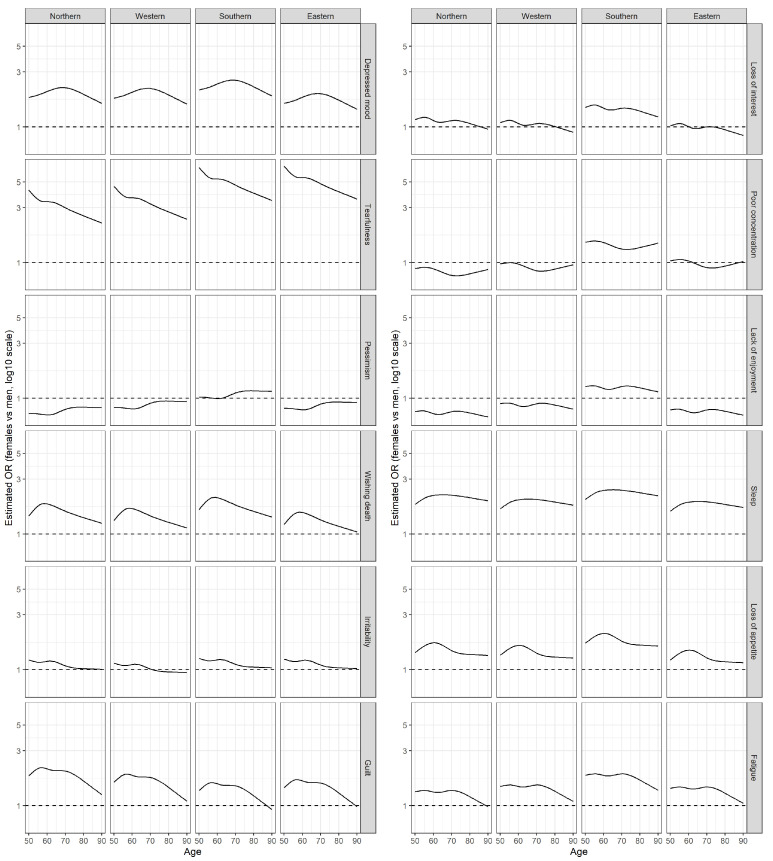
Estimated OR for females versus males for symptoms, by age and region. Estimates were obtained with from logistic regression models including two-way interactions between age, sex and region (base model) and additional risk factors (education, BMI, current smoking, presence of chronic disease, vigorous physical activity, living arrangement; adjusted model); age was modelled using restricted cubic splines (rcs). See Table 3 for sample sizes within each region and by sex, and for descriptive statistics.

The percentage of participants with depressive symptoms in the subscales of EURO-D ranged from 7% (wishing death) to 39% (depressed mood) (Appendix C Table A2). Overall, the highest prevalences were usually observed in the Southern region (loss of interest, poor concentration, lack of enjoyment, tearfulness) and the Eastern region (depressed mood, pessimism, sleep, irritability, fatigue) (Figure 4 and Figure 5). Women were more likely to report depressed mood, tearfulness, trouble with sleeping, and fatigue. Sex differences were consistent across regions, but diminished with age (Appendix C Figure A3).

## 4. Discussion

### 4.1. Data Organization

A comprehensive data quality framework for research data collections in observational health research includes integrity (compliance with pre-specified formats and structures), completeness, consistency (errors or inadmissible values, or contradictions) and accuracy (univariate and multivariate distributions) [21]. Data pre-processing steps and initial data analysis are crucial to understand data properties, their suitability for statistical models and interpretation of findings [20]. This includes efficient use of data cleaning steps to identify inconsistencies and make corrections, if possible, in a reproducible manner. A plan or workflow for data pre-processing should be included in study protocols [22]. The structure of the SHARE data is complex and we provide vignettes with R code to considerably reduce the time needed to prepare an analysis-ready dataset.

### 4.2. Data Application: Depression in Older Adults

We illustrated the use of a step-by-step approach in a case study where the aim was to develop a predictive model for clinical depression. Data filtering tools keep track of the number of participants excluded from the study in each step of applying exclusion criteria. Exploring the extent, pattern, and reasons for missing values can uncover systematic study design issues that can be considered in the analysis. We recommend that missing data be routinely examined at baseline, by wave and by type of interview. Data management may reveal that survey questions changed over time resulting in transformation of variables or defining new variables to account for such changes or data properties. Our example indicates that care is needed when SHARE data or other longitudinal data is used. Differences between questionnaires are well documented in the metadata of the SHARE project, but may be overlooked, if many variables are considered in the analysis. Many research projects include such data management tasks. Our systematic approach provides some guidance for data pre-processing and for initial data analysis. This approach and the reproducible R code provided can be adapted to other examples, saving substantial time to researchers.

### 4.3. Age and Sex Differences in Prevalence of Depression

Depression is a major cause of disability especially at older ages and there are sex differences. The prevalence of depression varies across European countries, ranging from 17% to 40%, but, as previously observed, the sex differences are remarkably consistent across countries [16]. Women report more depressive symptoms than men across ages 50 to 90, an almost 2-fold difference after adjusting for other variables. Our results confirmed previous findings, namely less education, living alone, smoking, chronic diseases, lack of vigorous physical activity, or limitations in instrumental daily activities are associated with a higher prevalence of depressive symptoms [17].

The probability of depression varies with age in a nonlinear fashion throughout the lifespan, but an age-associated pattern has not been established in older ages [17]. Some studies concluded that a decrease in depressive disorders in older age has been observed in European countries [23], while other studies observed an increase in depressive symptoms with age, but also noted variations in prevalence of depressive symptoms between study centres, and that these were not always consistent with levels of depressive disorder [24,25]. The mixed messages about age-associated increase or decease in depression with older age are partially due to the definition of depression, clinically diagnosed or depressive symptoms, or collected via surveys or across centers. It also depends on the age ranges included in the study. The choice of model with linear or non-linear age effect and the selection of covariates to be included in these models account for some of the differences.

In our study we considered depressive symptoms defined by a cutoff point of EURO-D ≥4 to indicate clinical depression [9]. Differences in health care access, socioeconomic inequalities, education levels could in part explain variation across countries. The relevance of older age as an explanatory variable for the prevalence of depression may depend on other explanatory variables used in statistical models and whether age is included as a linear or non-linear variable. Without adjusting for other factors in a linear model, the estimated probability of depression increases with age and there are large differences between countries. After adjusting for education, health and lifestyle factors, there is at first a decrease in the probability of depression after age 50 and then it stays constant or slightly increases in some regions. In Southern European countries, depression increases more steeply at older ages. Thus, it is important consider the geographic region, demographics, and functional form of explanatory variables when comparing studies on depression.

The analysis of individual depressive symptoms identified risk factors associated with subscales of EURO-D. Sex differences were observed for depressed mood, tearfulness, sleep problems and fatigue.

Strengths and limitations. This is a cross-sectional study and thus longitudinal changes cannot be evaluated. While missing values can be a challenge in survey collections, with a thorough data cleaning process we were able to leverage information across waves and thus reduce the proportion of missingness. Sampling procedures in different countries may influence the results regarding the estimated prevalence of depression that can lead to an underestimate of the variation. We addressed this by conducting sensitivity analyses with weighted and unweighted models. Testing associations across multiple outcomes such as the individual items of the EURO-D scale can increase the chances of spurious results. It is reassuring that we saw similar patterns in different models and in the analysis of subsamples for single waves.

## 5. Conclusions

Our framework contains steps and tools to facilitate working with SHARE data. The analysis and R code is fully reproducible. Our hope is that this paper will empower researchers to use this rich, multidisciplinary resource available for scientific studies. In particular, we illustrate the use of this data in modeling the probability of depression across geographic regions and discuss the influence of modeling choices on findings.

## Figures and Tables

**Table 1:** Number of participants by region and Wave of first interview.

First Interview	Northern	Western	Southern	Eastern
Wave 1	4268	13,940	6861	0
Wave 2	1821	4489	2638	4762
Wave 4	498	13,149	4695	15,529
Wave 5	4320	9178	4667	2439
Wave 6	352	1875	3882	4698
Wave 7	0	1	5	2

**Table 2:** Descriptive statistics (*n* = 104,069). Md (Q1, Q3) represent the median, lower quartile and the upper quartile for continuous variables. Numbers after percentages are frequencies. Countries were categorized into four regions: Northern Europe (Denmark and Sweden), Western Europe (Austria, Germany, France, the Netherlands, Switzerland, Belgium, Ireland and Luxembourg), Southern Europe (Spain, Italy, Greece and Portugal) and Eastern Europe (Czech Republic, Poland, Hungary, Slovenia, Estonia and Croatia). PA: Physical activity, IADL: Instrumental activities of everyday life index, EURO-D: 12-item Europe depression scale.

	Overall (*n* = 104,069)		Northern (*n* = 11,259)		Western (*n* = 42,632)		Southern (*n* = 22,748)		Eastern (*n* = 27,430)	
	Male	Female	Male	Female	Male	Female	Male	Female	Male	Female
	*n* = 47,887	*n* = 56,182	*n* = 5385	*n* = 5874	*n* = 19,873	*n* = 22,759	*n* = 10,672	*n* = 12,076	*n* = 11,957	*n* = 15,473
Age Md (Q1, Q3)	63 (56, 71)	62 (55, 71)	63 (56, 71)	62 (55, 70)	62 (55, 70)	62 (55, 70)	63 (56, 71)	62 (55, 71)	63 (57,71)	63 (56, 72)
Age groups: 50–59	39% (18,529)	41% (22,775)	38% (2056)	42% (2438)	41% (8231)	43% (9782)	37% (3947)	40% (4870)	36% (4295)	37% (5685)
60–69	34% (16,082)	31% (17,627)	34% (1825)	33% (1925)	33% (6466)	30% (6866)	33% (3551)	31% (3784)	35% (4240)	33% (5052)
70–80	22% (10,676)	22% (12,330)	22% (1187)	20% (1199)	21% (4173)	21% (4712)	24% (2537)	22% (2681)	23% (2779)	24% (3738)
80+	5% (600)	6% (3450)	6% (317)	5% (312)	6% (637)	6% (741)	6% (637)	6% (741)	5% (643)	6% (998)
Education: Low	38% (18,000)	46% (25,811)	30% (1641)	33% (1956)	28% (5655)	40% (9082)	66% (7085)	73% (8814)	30% (3619)	39% (5959)
Medium	39% (18,693)	35% (19,785)	38% (2059)	31% (1836)	42% (8317)	38% (8732)	19% (2072)	17% (2028)	52% (6245)	46% (7189)
High	23% (11,004)	18% (10,332)	31% (1651)	35% (2042)	29% (5797)	21% (4822)	14% (1479)	10% (1183)	17% (2077)	15% (2285)
Other	0% (190)	0% (254)	1% (34)	1% (40)	1% (104)	1% (123)	0% (36)	0% (51)	0% (16)	0% (40)
BMI Md (Q1, Q3)	27 (24, 29)	26 (23, 29)	26 (24, 28)	25 (23, 28)	26 (24, 29)	25 (23, 29)	27 (25, 29)	26 (23, 29)	27 (25, 30)	27 (24, 30)
Living alone: Yes	19% (9253)	34% (19,189)	21% (1114)	30% (1764)	21% (4269)	35% (7891)	14% (1513)	29% (3505)	20% (2357)	39% (6029)
Vigorous PA: >1 a week	41% (19,449)	32% (17,897)	50% (2716)	41% (2432)	43% (8496)	35% (8000)	32% (3461)	25% (3042)	40% (4776)	29% (4423)
Once a week	14% (6519)	14% (8094)	14% (732)	15% (874)	14% (2856)	15% (3341)	12% (1264)	14% (1681)	14% (1667)	14% (2198)
1–3 times a month	10% (4664)	10% (5339)	9% (461)	8% (448)	8% (1684)	7% (1677)	11% (1144)	13% (1517)	11% (1375)	11% (1697)
Hardly ever, or never	36% (17,255)	44% (24,852)	27% (1476)	36% (2120)	34% (6837)	43% (9741)	45% (4803)	48% (5836)	35% (4139)	46% (7155)
Current smoking: Yes	24% (11,313)	17% (9311)	20% (1055)	21% (1213)	22% (4399)	16% (3748)	25% ( 2656)	14% (1738)	27% (3203)	17% (2612)
Chronic diseases: 0	47% (22,270)	49% (27,346)	49% (2620)	52% (3071)	49% (9716)	53% (12,088)	49% (5194)	51% (6132)	40% (4740)	39% (6055)
1	34% (16,250)	34% (18,918)	33% (1758)	33% (1917)	33% (6605)	33% (7398)	34% (3664)	34% (4090)	35% (4223)	36% (5513)
2+	20% (9367)	18% (9918)	19% (1007)	15% (886)	17% (1814)	15% (1854)	17% (1814)	15% (1854)	25% (2994)	25% (3905)
IADL: 1+	11% (5117)	18% (10,052)	8% (446)	14% (806)	10% (1953)	17% (3793)	10% (1051)	18% (2226)	14% (1667)	21% (3227)
EURO-D ≥4	19% (9167)	34% (18,845)	12% (646)	23% (1347)	17% (3460)	30% (6864)	21% (2189)	39% (4724)	24% (2872)	38% (5910)

**Table 3:** Estimated OR (with 95% CI and p-value) obtained from logistic regression models including two-way interactions between age, sex and region and additional risk factors (education, BMI, current smoking, presence of chronic disease, vigorous physical activity, living arrangement; adjusted model); age was modelled using restricted cubic splines (rcs). Estimates for age, sex and region and their two-way interactions are omitted but presented graphically in Figure 2; see Table 1 for sample sizes within each region and by sex, and for descriptive statistics.

	OR	95% CI LL	95% CI UL	*p*-Value
BMI (10 units)	1.02	0.99	1.06	0.17
Education: Low	1			
Medium	0.75	0.72	0.77	<0.01
High	0.64	0.61	0.66	<0.01
Other	0.98	0.79	1.22	0.87
Current smoking: Yes	1.24	1.20	1.29	<0.01
Chronic diseases: 0	1			
1	1.45	1.40	1.50	<0.01
2+	2.52	2.41	2.62	<0.01
Vigorous PA : >1 a week	1			
Once a week	1.03	0.98	1.08	0.19
1–3 times a month	1.11	1.05	1.17	<0.01
Hardly ever, or never	1.78	1.72	1.85	<0.01
Living alone: Yes	1.41	1.37	1.46	<0.01

## Data Availability

This study used data from the Survey of Health, Ageing and Retirement in Europe, which is freely available to academic researchers. http://www.share-project.org accessed on 15 July 2021. Vignettes and code are available on the Open Science Framework platform [13].

## Abbreviations

   The following abbreviations are used in this manuscript:

SHARE	Survey of Health, Ageing and Retirement in Europe
IDA	Initial Data Analysis
EURO-D	Europe depression scale

## Appendix A. SHARE Data Organization

Data can have one of two formats: long format (for specific waves and easySHARE) with multiple rows per individual corresponding to different measurement times, or wide format for data across waves with one row per individual and variables are spread across different columns. Data for the same individual from different modules or waves can be merged with a unique identifier, mergeid with format CC-hhhhhh-rr, where CC is the country code, hh is the household code and rr is the responder’s identifier within the household.

Data folders for each wave contain files for each of the modules included in this wave. Selected household members provide responses regarding family, financial or household, on behalf of other participants. They can be identified using the fam_resp, fin_resp and hou_resp variables in the CV_R or technical variables (TV) modules. The list of modules with an indication of responders is given in Table A1. Types of interviews are are baseline or longitudinal questionnaires (indicated by the mn101_ variable in the CV_R module). Some questions are asked only at baseline. It is important to recognize that some questions differ in the baseline vs. longitudinal questionnaires, despite being stored with the same variable name, examples are given below. In wave 3 and partly in wave 7 a different questionnaire (retrospective SHARELIFE) was used to collect retrospective information about participants. The modules are described in detail in the SHARE release guide that also includes codes for missing values [26].

SHARE provides transformations of some variables to facilitate the comparison across-countries. For example, the depression scale EURO-D is provided summing the scores from the 12 questions that constitute it; education is reported using international standardized codes (ISCED). The modules including generated variables have names starting with gv_ and they appear within the wave directories.

The content of an end-of-life interview, conducted with a proxy-respondent in case a participant died, is available in the (xt module). The retrieval of information about deaths and about overall participation in the study is greatly simplified by using the information provided in the gv_allwaves_cv_r cross-wave module, which organizes data in wide format and data cleaning was performed.

**Table A1:** List of the modules and type of responders; modules starting with gv are generated variables modules.

Module	Description	Responder	Waves (1–7)
AC	Activities		all
AS	Assets financial		all
BR	Behavioural risk		all
BS	Blood sample		W6
CF	Cognitive function		all
CH	Children	family	W2, W5
CM	Household demographics		all
CO	Consumption household		all
CS	Chair stand		all
CV_R	Coverscreen for individuals		all
DN	Demographics and network		all
EP	Employment and pensions		all
EX	Expectations		all
FT	Financial transfers	financial	all
GS	Grip strength		all
HC	Health care		all
HH	Household income	household	all
HO	Housing household		all
IT	Use of IT technology		W5-7
IV	Interviewer observations	inteviewer	all
LI	Linkage to pension data		all
MC	Mini-childhood		W5
MH	Mental health		all
PF	Peak flow		W2-3, W6
PH	Physical health		all
SC	Cover screen		all
SN	Social networks		W4, W6
SP	Social support	partly family for the couple	all
TV	Technical variables		all
WS	Walking speed		W1-2
XT	End of life	proxy	W2-7
gv_big5	Big Five personality traits		W7
gv_children	Combined children information		W6-W7
gv_dbs	Dried Blood Spots		W6
gv_deprivation	Indices for material and social deprivation		W5
gv_grossnet	Net income measures		W1
gv_health	Physical and mental health		W1-2, W4-7
gv_housing	Housing and NUTS codes		W1-2, W4-7
gv_imputations	Imputed values		W1-2, W4-7
gv_isced	International Standard Classification of Education		W1-2, W4-7
gv_isco	Classification of occupations		W1
gv_networks	Information on social networks		W4, W6
gv_ssw	Social security wealth		W4
gv_weights	Weights		all

## Appendix B. Data Import with R

After downloading the SHARE data in STATA format, they can be imported into R [6]. The size of the complete unzipped data in STATA format is about 5 GB. Files are named to describe the wave/release/module: for example, the sharew1_rel7-1-0_ph.dta file stored in the directory for the first wave refers to the module on physical health, named PH, collected in wave 1, from the 7-1-0 release.

To use the R function f_readshare the original naming of the files should be maintained and the files should be unzipped in a local folder. The user specifies from which modules, and possibly waves, she wants to download the data; to avoid downloading data that are not of interest, the user can further restrict the download to a subset of the countries, and/or a subset of the variables within modules. Data from wave-specific and cross-wave modules are downloaded in separate elements of the output of the function: wave-specific modules are stored together in a long format R data frame, while the cross-wave modules are saved in a list and in wide format. The code for using this function are reported in reproducible, open access vignettes ShareDataRetrieval.html [13].

Given the complexity of the variable structure of the SHARE project, users should prepare a data dictionary with a list of variables of interest and their corresponding SHARE modules. An example is available in the file SHARE_Metadata_OSF.xlsx [13].

## Appendix C. Data Application: Depression in Older Adults

### Appendix C.1. Analysis of the 12-Item EURO-D Scale

In this section we present the additional results obtained modelling depression.

In Figure A1 we compared the odds for depression of women versus men at different ages and in different regions. The estimates obtained from the adjusted model indicated a decrease of sex OR with age. The OR in the Southern region were substantially larger than in the other regions.

In Figure A2 we compared the estimated probabilities of depression obtained modelling age using restricted cubic splines and with a linear effect of age.

Adjusted models used know risk factors for depression encompassing demographics, chronic diseases, lifestyle factors and health behaviors as described in the Methods section.

Details can be found in Part2-DataAnalysis.html [13].

### Appendix C.2. Analysis of Single Items of EURO-D

In this section we present results obtained from the analysis of all single symptoms in the EURO-D scale.

The percentage of participants with depressive symptoms overall and across regions is shown in (Table A2).

In Figure A3 we compared the odds for selecting a symptom of women versus men at different ages and in different regions. The estimates obtained from the adjusted model indicated a decrease of sex OR with age. The OR in the Southern region were substantially larger than in the other regions for many symptoms.

**Table A2:** Descriptive statistics for the prevalence of symptoms. Numbers after percentages are frequencies, *n* indicates the sample size. Countries were categorized into four regions: Northern Europe (Denmark and Sweden), Western Europe (Austria, Germany, France, the Netherlands, Switzerland, Belgium, Ireland and Luxembourg), Southern Europe (Spain, Italy, Greece and Portugal) and Eastern Europe (Czech Republic, Poland, Hungary, Slovenia, Estonia and Croatia).

	Overall (*n* = 104,069)		Northern (*n* = 11,259)		Western (*n* = 42,632)		Southern (*n* = 22,748)		Eastern (*n* = 27,430)	
	Male	Female	Male	Female	Male	Female	Male	Female	Male	Female
	*n* = 47,887	*n* = 56,182	*n* = 5385	*n* = 5874	*n* = 19,873	*n* = 22,759	*n* = 10,672	*n* = 12,076	*n* = 11,957	*n* = 15,473
euro1: Depressed mood	30% (14,400)	47% (26,538)	23% (1255)	38% (2260)	31% (6108)	47% (10,771)	27% (2873)	47% (5627)	35% (4164)	51% (7880)
euro2: Pessimism	18% (8372)	18% (10,309)	6% (326)	5% (306)	12% (2429)	12% (2798)	22% (2392)	25% (3008)	27% (3225)	27% (4197)
euro3: Wishing death	5% (2499)	9% (4936)	3% (143)	5% (269)	6% (1119)	9% (2071)	4% (463)	8% (990)	6% (774)	10% (1606)
euro4: Guilt	7% (3113)	10% (5810)	6% (327)	12% (680)	6% (1213)	10% (2293)	6% (649)	9% (1036)	8% (924)	12% (1801)
euro5: Sleep	25% (12,198)	41% (23,138)	23% (1222)	38% (2241)	26% (5073)	40% (9134)	20% (2180)	37% (4508)	31% (3723)	47% (7255)
euro6: Loss of interest	8% (3760)	10% (5587)	6% (303)	7% (402)	6% (1181)	7% (1602)	11% (1214)	16% (1986)	9% (1062)	10% (1597)
euro7: Irritability	27% (13,107)	29% (16,463)	21% (1111)	23% (1339)	26% (5099)	26% (6012)	29% (3090)	31% (3798)	32% (3807)	34% (5314)
euro8: Loss of appetite	6% (3044)	10% (5461)	5% (262)	8% (443)	6% (1158)	9% (1997)	7% (773)	12% (1490)	7% (851)	10% (1531)
euro9: Fatigue	29% (13,832)	39% (21,802)	29% (1572)	36% (2094)	26% (5218)	35% (7940)	27% (2903)	40% (4851)	35% (4139)	45% (6917)
euro10: Poor concentration	17% (8021)	19% (10,721)	12% (652)	11% (648)	17% (3430)	18% (4148)	21% (2208)	28% (3381)	15% (1731)	16% (2544)
euro11: Lack of enjoyment	13% (6234)	13% (7552)	9% (504)	8% (452)	12% (2334)	12% (2665)	16% (1756)	21% (2483)	14% (1640)	13% (1952)
euro12: Tearfulness	11% (5440)	36% (20,045)	10% (559)	28% (1646)	13% (2645)	36% (8253)	12% (1259)	41% (4966)	8% (977)	34% (5180)

Details can be found in Part3-SubscalesAnalysis.html [13].

## Appendix D. Sampling Design and Weights

The sampling designs differ substantially across participating countries, from simple random sampling to multistage designs, depending on the sampling frame available in each country; SHARE documentation provides detailed descriptions of the methods used in each country [11]. The information provided in the gv_weights modules summarize the sampling design by providing the strata (stratum1 and stratum2) and primary sampling units (psu), when they were used; study design weights (dw) that take selection probabilities into account are also provided, but their usage is discouraged, due to unit non-response and sample attrition at each follow-up. Instead calibrated weights are advocated for inference to the target population (cciw for individuals and cchw for households) as these are design weights re-weighted to compensate for unit-non response.

Longitudinal weights (gv_weights_longitudinal modules) are also available. The target population surviving up to the time is considered, to take sample attrition due to mortality into account. The weights are provided for paired comparisons of adjacent waves or for the analysis of all available waves and stored in the gv_weights_longitudinal modules.

We used the survey R package [27,28] to take study design and weights into account. We considered stratum1 as the stratification variable (imputing a single strata in countries that did not use stratification), and psu as the primary sampling unit used for clustering (using a different psu for each participant in the countries where clustering was not used) and used the cross-sectional calibrated weights provided by SHARE. We obtained weighted estimates of prevalence of depression and estimated weighted logistic regression models. We provide the results for each wave for the 20 countries, if the country was included in the wave. We also performed a weighted analysis of the interviews obtained in the wave when a country was first included in the SHARE study. The results were compared to unweighted analyses. The aim of these analyses was to explore the sensitivity of the results to omitting weights as recommended in [28] (Section 5.3).

Weighted and unweighted analyses yielded similar odds ratios for the risk factors. In some waves/regions the weighted prevalence of depression was higher than the unweighted; the biggest differences were observed in the Eastern region in waves 2 and 6. Detailed code and results are included in Part4-Samplingweights.html [13].
